# A versatile kinase mobility shift assay (KiMSA) for PKA analysis and cyclic AMP detection in sperm physiology (and beyond)

**DOI:** 10.3389/fcell.2024.1356566

**Published:** 2024-02-20

**Authors:** Analia G. Novero, Catalina Curcio, Tomás J. Steeman, Andres Binolfi, Diego Krapf, Mariano G. Buffone, Dario Krapf, Cintia Stival

**Affiliations:** ^1^ Cell Signal Transduction Networks, Instituto de Biología Molecular y Celular de Rosario (IBR) (CONICET-UNR), Rosario, Argentina; ^2^ Cellular-Structural Biology Lab, IBR (CONICET-UNR), Rosario, Argentina; ^3^ Department of Electrical and Computer Engineering, Colorado State University, Fort Collins, CO, United States; ^4^ Instituto de Biología y Medicina Experimental (IBYME-CONICET), Ciudad Autónoma de Buenos Aires, Argentina

**Keywords:** protein kinase A (PKA), sperm capacitation, kinase assay, cAMP, fertility, kemptide kinase activity, non-radioactive assay, phosphorylation

## Abstract

The cAMP-dependent protein kinase (PKA) is one of the most extensively distributed kinases among intracellular signal cascades, with a pivotal role in the regulation of various processes, including the capacitation of sperm cells. Traditional assessments of PKA activity relies on the utilization of [γ-^32^P] ATP and the Kemptide substrate. This methodology presents several major drawbacks, including high-costs and health risks derived from the manipulation of radioactive isotopes. In this work we introduce an enhanced non-radioactive assay for quantifying PKA activity, termed KiMSA which relies on the use of a fluorescent-labeled Kemptide (Kemptide-FITC). Once the kinase reaction is terminated, the products can be easily resolved through electrophoresis on an agarose gel and quantified by fluorescence densitometry. We show that the KiMSA assay is suitable for purified PKA, and also to address both basal and capacitation induced PKA activity in mouse sperm cells. Furthermore, the assay enables monitoring the inhibition of PKA with inhibitors such as sPKI and H-89 in live cells. Therefore, the experimental and optimal assay conditions are set so that the KiMSA assay can be used to either assess *in vitro* as well as *in vivo* PKA activity in sperm cells. Finally, this method allows for measurement of cAMP concentrations, rendering a versatile technique for the study of cAMP/PKA pathways.

## 1 Introduction

For mammalian sperm to fertilize an oocyte, they first need to undergo a process known as sperm capacitation (Chang, 1951; Austin, 1952), which can be achieved both *in vivo* during their transit through the female reproductive tract, or *in vitro* by incubating the sperm cells in a defined capacitating medium (Stival et al., 2016). One of the first events during capacitation is the activation of the Protein Kinase A (PKA) also known as cAMP-dependent kinase A, which phosphorylates proteins on either Ser or Thr residues (Baro Graf et al., 2020; Balbach et al., 2021). Early activation of PKA in sperm cells regulates several downstream signalling cascades involved in sperm capacitation (Stival et al., 2016). In addition, a wide body of literature initiated by the pioneer work of Walsh et al. (1968) on phosphorylation related to cAMP-dependent protein kinase activity, indicates that PKA is responsible for phosphorylating a broad array of targets, and considered as an essential regulator of many signalling events in somatic cells (Carnegie et al., 2009). The PKA holoenzyme is constituted by two regulatory subunits (PKAR) bound to two catalytic subunits (PKAc), keeping the enzyme in an inactive state (Akamine et al., 2003; Zhang et al., 2012; Taylor et al., 2013).

Sperm capacitation is achieved *in vitro* upon incubation of sperm in media containing bicarbonate among other standard components of culture media. Bicarbonate stimulates a soluble adenylate cyclase (sAC) present in sperm (Balbach et al., 2021), triggering an increase of intracellular cAMP. When sperm are incubated under capacitating conditions, the increase of intracellular cAMP induces a conformational change on the regulatory subunits of PKA that releases PKAc into an active state (Taylor et al., 2013; Baro Graf et al., 2020). Since PKA is a central regulator of capacitation and, in turn, of male fertility, investigating its activation is highly relevant in the reproductive field. At present, the predominant approach for assessing direct PKA activity takes advantage of the availability of a specific synthetic substrate for PKA called Kemptide, which comprises an 8-aminoacid sequence (LRRASLGK) containing the consensus phosphorylation site for PKA (RRXS/T) (Kemp et al., 1977; Araujo et al., 2016). By incubating the enzyme in the presence of radioactive [γ-32P]-ATP, the activity of PKA can be measured by determining the amount of radioactive phosphate transferred to the Kemptide (Wertheimer et al., 2013; Stival et al., 2018; Stival et al., 2020). However, this methodology has several disadvantages related to ^32^P, including health risks, high costs, short half-life of radioisotopes, and the need of trained personnel and specialized facilities. To overcome these drawbacks, Lutz and co-workers proposed the use of fluorescence instead of radioactivity to detect a phosphorylated substrate (Lutz et al., 1994). Phosphorylation of Kemptide by PKA introduces two negative charges to the peptide, resulting in a shift of its net charge. The extra negative charges cause phosphorylated Kemptide to further migrate towards the anode when subjected to agarose gel electrophoresis, allowing the separation of the non-phosphorylated from the phosphorylated version (Lutz et al., 1994; Araujo et al., 2016). This approach using a fluorescent-labelled Kemptide yielded similar results than the standard assay employing [γ32P]-ATP (MacAla et al., 1998) and has been later applied to assess the *in vitro* activity of PKA purified from pig heart (Araujo et al., 2016).

Here, we have improved this methodology by developing a Kinase Mobility Shift Assay (called KiMSA), which enhances previous methodologies so that it can be used to 1) study *in vitro* the activity of either purified or recombinant PKAc, and 2) specifically reflect *in vivo* status of PKA in sperm extracts. This procedure can be used to differentiate between non-capacitated and capacitated sperm PKA activity status. Furthermore, we exploit the cAMP dependent activity of PKA holoenzyme, to put forward a third application of KiMSA to directly assess the concentration of cAMP in cell extracts.

## 2 Materials and methods

### 2.1 Chemical and reagents

Chemicals were obtained from the following sources: Bovine Serum Albumin (BSA) fatty acid-free (#A7906), adenosine 3-phosphate (ATP, #A7699), Cyclic AMP (cAMP, #A9501), from Sigma-Aldrich. Myristoylated PKI 14–22 amide (sPKI, #2546) from Tocris. PhosStop protease inhibitor (#4906837001) and cOmplete EDTA-free protease inhibitor cocktail (#4693132001) from Roche. Rabbit monoclonal anti-phospho-PKA substrates (⍺-pPKAs, clone 100G7E, #9624S) from Cell Signalling Technology (Danvers, MA). Horseradish peroxidase-conjugated -mouse and -rabbit IgG, and anti-mouse (#211-032-171) were purchased from Jackson ImmunoResearch Laboratories (West Grove, PA). Anti-tubulin beta antibody was purchased from DSHB (clone E7, AB_2315513). The traditional non-fluorescent Kemptide (sequence LRRASLG) was purchased from AnaSpec (#22594) while the fluorescent version of the Kemptide (termed “Kemptide-FITC”) was custom-synthesized by Biomatik with the sequence LRRASLGK-FITC (FITC fluorophore is conjugated to the C-terminal of the peptide sequence). All other chemicals were purchased from Cayman Chemicals (Ann Arbor, MI).

cAMP, DTT, ATP, Kemptide, Kemptide-FITC and inhibitor dilutions were prepared fresh the day of the experiment. sPKI was dissolved in dimethylsulfoxide (DMSO) which was also included as a vehicle control when needed. Kemptide-FITC should always be kept in dark to prevent quenching of the fluorophore.

### 2.2 Purification of the recombinant PKA catalytic subunit alpha (recPKAc)

A starter Luria Bertari (LB) broth culture supplemented with 100 μg/mL ampicillin was inoculated with *Escherichia coli* BL21 previously transformed with the pET15b_Prkaca vector. pET15b PKA Cat was a gift from Susan Taylor (Addgene plasmid # 14921; http://n2t.net/addgene:14921; RRID: Addgene_14921). This plasmid codifies for the PKA catalytic subunit alpha of *Mus musculus* fused to 6xHis tag at the N-terminal. Culture was grown overnight at 37°C and then transferred to 1,000 mL of LB medium supplemented with 100 μg/mL ampicillin. The culture was allowed to grow until an optical density of 0.4 and 0.5 mM IPTG was added to induce protein expression. Overexpression was done overnight at 18°C. Cells were harvested by centrifugation at 5,000 g, for 30 min at 4°C and the pellet resuspended in 20 mL of cold Bacterial Lysis Buffer (20 mM Tris-HCl pH = 8.0, 50 mM KH_2_PO_4_, 100 mM NaCl, 5 mM β-mercapthoethanol, 1 mM PMSF). Cells were sonicated on ice 10 times for 7 s at 30.5% sonicator power (Branson, U.S.A.). Cell debris was removed by centrifugation at 9,500 g for 30 min at 4°C. For every 2 mL of the supernatant, 250 μL of Dynabeads™ His-Tag Isolation and Pulldown (Invitrogen™, Carlsbad, CA, USA) were added, and the mixture was agitated at 4°C for 30 min. The flow-through was removed and the Dynabeads™ were washed with 20 mL of Bacterial Lysis Buffer. The attached PKAc-His protein was sequentially eluted with 2 mL of Bacterial Lysis Buffer supplemented with either 25, 50, 250 and 1,000 mM imidazole. All eluted fractions were saved and analyzed by SDS-PAGE to trace the PKAc-His rich fractions. The purified enzyme was concentrated, and the buffer was changed to 20 mM KH_2_PO_4_, 100 mM NaCl, 10 mM MgCl_2_, pH = 7.0, using a 10 K MWCO filter Pierce™ Protein Concentrators (Fisher Scientific).

Quantification was performed by absorbance at 280 nm, considering an extinction molar coefficient (ε) of 53860 as estimated by the “ProtParam” tool of the free-access software “Expasy” (www.expasy.org) for the specific recombinant protein sequence.

### 2.3 Animals

C57BL/6 male mature (10–15 weeks old) male mice were used. In all cases, mice housing and all experimental procedures were conducted in accordance with Animal Care and Use Committee of the Facultad de Ciencias Bioquímicas y Farmacéuticas de Rosario (UNR), Argentina (protocol approved #434/2023). The Guide for Care and Use of Laboratory Animals approved by the National Institutes of Health (NIH) was strictly met.

### 2.4 Preparation of non-capacitated and capacitated mouse sperm extracts

Cauda epididymal mouse sperm were collected from adult male mice (10–13 weeks old). Each minced cauda epididymis was placed in 600 μL of HEPES-buffered TYH medium (H-TYH) containing 119.3 mM NaCl, 4.7 mM KCl, 1.2 mM KH_2_PO_4_, 1.2 mM MgSO_4_, 5.6 mM glucose, 0.5 mM sodium pyruvate, 1.7 mM Ca^2+^, and 20 mM HEPES (pH 7.3), accounting for non-capacitating medium (“NC medium”). After 15 min of incubation at 37°C (“swim-out”), epididymides were removed and the suspension was adjusted with NC medium to a final concentration of 1–2 x10^6^ cells/mL. For capacitation, BSA and NaHCO_3_ were added to final concentrations of 5 mg/mL and 20 mM respectively (“CAP medium”). In the experiments where endogenous PKA activity was assessed, sperm were incubated in either NC or CAP medium at 37°C for 15 min, sufficient to trigger PKA activation (Baro Graf et al., 2020; Balbach et al., 2021). Note that unless otherwise stated, 7.5 × 10^6^ sperm/mL (3 × 10^6^ sperm in 400 µL) were treated in each condition (so that every 10 µL of total sperm extract added to the PKA kinase reaction would represent an extract from 300,000 sperm cells, see “PKA kinase reaction”). In cases where PKA inhibitors (sPKI or H89) were used, sperm were pre-incubated with the respective reagents in NC medium for 10 min prior to the incubation in the CAP medium further supplemented with the inhibitors.

For preparation of total sperm extracts used to quantify endogenous PKA activity in cells incubated in either NC or CAP media, sperm were pelleted by centrifugation at 1,000 *g* for 3 min at RT, and a precise volume of the supernatant was discarded from each reaction tube, leaving 30 µL of supernatant above the pelleted cells. Cells were re-suspended in 70 µL of cold Triton Lysis Buffer (final concentrations 25 mM Tris-HCl, 150 mM NaCl, 1X EDTA-free protease inhibitor mixture, 1X PhosSTOP cocktail inhibitor, 1% Triton X-100, 10 mM DTT, pH 7.4) to achieve a final volume of 100 µL. Note that re-suspending cells to equal final volumes in all treatments is critical to get consistent results. If these steps are not done carefully, differences in PKA apparent activity may arise due to different dilution factors of the sperm extracts. To achieve cell lysis, sperm were incubated for 30 min on ice with frequent gentle pipetting. Samples were then either immediately used in PKA kinase reactions or nitrogen-frozen and stored at −80°C for future use in 50% Glycerol.

### 2.5 Preparation of sperm insoluble fraction as source of PKA for cAMP assay

For the preparation of insoluble sperm extracts, used as source of PKA holoenzyme to measure cAMP concentrations, sperm were processed as detailed elsewhere (Wertheimer et al., 2013). In brief, sperm cells were directly diluted in NC medium after swim-out to achieve a final concentration of 7.5 × 10^6^ sperm cells/mL (i.e., 3 × 10^6^ sperm in 400 µL). Cells were pelleted by centrifugation at 10,000 *g* for 3 min at RT and 30 µL of supernatants were left before adding 70 µL of ice-cold Triton Lysis Buffer (final concentrations 25 mM Tris-HCl, 150 mM NaCl, 1X EDTA-free protease inhibitor mixture, 1X PhosSTOP cocktail inhibitor, 1% Triton X-100, 10 mM DTT, pH 7.4) to a final volume of 100 µL. This total extract was further centrifuged at 10,000 g for 10 min at 4°C, and the pelleted cells re-suspended in a final volume of 100 µL of ice-cold Triton Lysis Buffer to obtain the insoluble fraction that contains the PKA holoenzyme.

### 2.6 PKA kinase reaction

Each kinase reaction was made by mixing 5 µL of 5X Kinase Buffer (1 M Tris-HCl pH 7.4; 50 mM MgCl_2_; 1 mM ATP, 50 mM DTT, 5 X cOmplete EDTA-free protease inhibitor cocktail and 5X PhosSTOP); 2.5 µL of 0.4 µg/µL Kemptide-FITC (equivalent to 30 µM); 2.5 µL of 0.24 μg/μL non-fluorescent Kemptide (equivalent to 30 µM) and a variable volume (1–10 µL) of either total sperm extract or insoluble fraction (depending on the experiment). MiliQ water was added to achieve a final volume of 25 µL. Detection of NC and CAP status of PKA requires 10 µL of sperm total fraction, equivalent to 300,000 sperm cells. For cAMP determination assays, 10 µL of sperm insoluble fraction equivalent to 300,000 cells were used in each reaction.

For negative PKA activity control, Lysis Buffer replaced either sperm extract or recombinant PKAc, according to the experiment. For measurement of maximum PKA activity (used as positive activity control), 10 µL of either the CAP or the NC total sperm extract and 1 µM exogenous cAMP were added to the kinase reaction.

All reaction components were combined and kept on ice until incubated for 25 min at 37°C in the dark. Reactions were stopped by heating at 95°C for 1 min, before mixing with loading buffer 10X (63%–70% Glycerol; 1% Bromophenol blue; 5% Tween-20, 5 mM DTT). Finally, samples were centrifuged at 10,000 g for 1 min at 4°C and supernatants were kept on ice or stored at −80°C until assayed by agarose electrophoresis.

### 2.7 cAMP assay

Determination of cAMP concentration through PKA activity was performed by mixing the sperm insoluble fraction (as a source of the PKA holoenzyme) with both Kemptide and Kemptide-FITC, as described in the “PKA Kinase Reaction” section of Materials and Methods. Briefly, 10 µL of the insoluble sperm fraction equivalent to the protein content of 300 × 10^3^ cells were mixed with 5 µL of 5X Kinase Buffer (1 M Tris-HCl pH 7.4; 50 mM MgCl^2^; 300 µM ATP, 50 mM DTT, 5X cOmplete EDTA-free protease inhibitor cocktail and 5X PhosSTOP); 2.5 µL of 0.4 μg/μL Kemptide-FITC, 2.5 µL of 0.24 μg/μL Kemptide and 1–5 µL known amounts of cAMP for the standard curve (or unknow for cAMP determinations), and water as needed to complete a final volume of 25 µL. A standard curve using known concentrations of cAMP should be performed for each assay. Then, a plot correlating PKA activity with known cAMP concentrations on a logarithmic scale can be used to determine unknown cAMP concentrations by interpolation, as described previously (Wertheimer et al., 2013). Kinase reactions were started by incubating reaction mixtures for 25 min at 37°C. After the incubation period, reactions were stopped by 1 min at 100°C. Samples were centrifuged at 10,000 *g* for 1 min at 4°C and the supernatants were assayed by agarose electrophoresis.

### 2.8 Electrophoresis on agarose gels

Kinase reaction samples were loaded and subjected to electrophoresis (140 V for 40–60 min) on a horizontal 1.5% agarose gel in 50 mM Tris (pH 10.0). Note that the 50 mM Tris Buffer was used both for diluting the agarose and as the running electrophoresis buffer. When possible, an empty lane was left between samples to account for diffusion effects. Electrophoresis was stopped before the Bromophenol blue dye reached the bottom of the gel.

Non-phosphorylated and phosphorylated Kemptide-FITC signals were visualized by UV transillumination. Fluorescence was assessed with a Typhoon-FLA 700 spectrofluorometer under excitation/emission of 473/580 nm. Note that detection settings could be adapted to other image acquisition systems available.

### 2.9 Data analysis

Scanned images of gel electrophoresis were analysed with ImageJ (Schneider et al., 2012) to quantify band densitometries. Raw results were expressed in arbitrary fluorescence units (AFU) for non-phosphorylated (“Kemptide”) and for the phosphorylated Kemptide-FITC (“pKemptide”). For rapid assessment of PKA activity, the percentage of product generated (i.e., % phosphorylation) was directly calculated. For this, total fluorescence in a particular lane (i.e., Kemptide + pKemptide) was established as 100%. Then, the percentage of phosphorylated Kemptide in the gel could be calculated as: % pKemptide = pKemptide AFU /(pKemptide AFU+ non-pKemptide AFU)*100

For kinase activity determination, the following steps were taken:

1. Calculate loaded *p*moles of Kemptide in the gel. For example, considering a 60 μM Kemptide present in the Kinase Reaction, a gel loaded volume of 4 μL represents 240 *p*moles of total Kemptide.
2. Estimate the AFU for each pKemptide and Kemptide bands in all lanes. Add up these two values to obtain total Kemptide signal.
3. Calculate the amount of *p*moles of pKemptide on the gel by converting the AFU values for the pKemptide and considering the total amount of Kemptide loaded on the gel. Multiply this by loading ratio (*i.e.*, volume of Kinase Reaction/volume loaded in gel) to obtain the total amount of product (*i.e.*, “total pKemptide”) generated in the reaction tube.

Note 1 total pKemptide accounts for both non-fluorescent and fluorescent pKemptide in the Kinase Reaction tube.

Note 2 Since one phosphate is transferred from a molecule of ATP to a molecule of Kemptide, results are expressed as *p*moles of ATP instead of *p*moles of Kemptide so it can be directly compared with measures performed elsewhere.

4. Finally, the amount of ATP per unit time was calculated considering 25 min Kinase Reaction periods and normalized to the amount of either sperm extract or purified protein.

### 2.10 SDS-PAGE and immunoblotting

After incubation under the experimental treatments, sperm were collected by centrifugation at 800 *g* for 4 min at RT and washed twice in 700 μL of TBS. The cell pellet was re-suspended in Laemmli sample buffer (Laemmli, 1970) without β-mercaptoethanol, vortexed for 15 s and boiled for 5 min. After centrifugation at 13,400 *g* for 3 min, 5% β-mercaptoethanol was added to the supernatants and boiled for 5 min. Protein extracts equivalent to 1 × 10^6^ sperm/lane were subjected to SDS-PAGE and transferred to PVDF membranes (Bio-Rad) at 250 mA for 90 min on ice. Membranes were blocked with 3% BSA in TBS containing 0.1% Tween-20 (T-TBS).

Membranes were first developed using a 1/3,000 dilution of anti-pPKA substrates antibody in T-TBS containing 1% BSA. Secondary HRP-conjugated anti-IgG rabbit and anti-IgG mouse antibodies were diluted 1/10,000 and 1/20,000 respectively in T-TBS containing 1% fat-free milk and developed using an enhanced chemiluminescence detection kit (Biolumina, Kalium Tech, Argentina) according to manufacturer’s directions. PVDF membranes were stripped at 60°C for 15 min in 2% SDS, 0.74% β-mercaptoethanol, and 62.5 mM Tris (pH 6.5) and washed twice for 10 min each time in T-TBS. Then, membranes were developed using anti-β-Tubulin antibody diluted in 1% BSA in T-TBS in a 1/10,000 dilution.

### 2.11 Statistical analysis

Statistical analyses were performed using Prism 9.5.0 (GraphPad, Boston, MA, USA). All data are shown as mean ± SEM. Statistical significance between two groups was determined using two-tailed, paired t-tests, and statistical significance between multiple groups using matched one-way ANOVA with Tukey post-comparison tests. Significant differences are indicated as *p < 0.05, **p < 0.01, and ***p < 0.001.

## 3 Results

### 3.1 Linearity signal of the non-phosphorylated Kemptide-FITC on agarose gels

**FIGURE 1:**
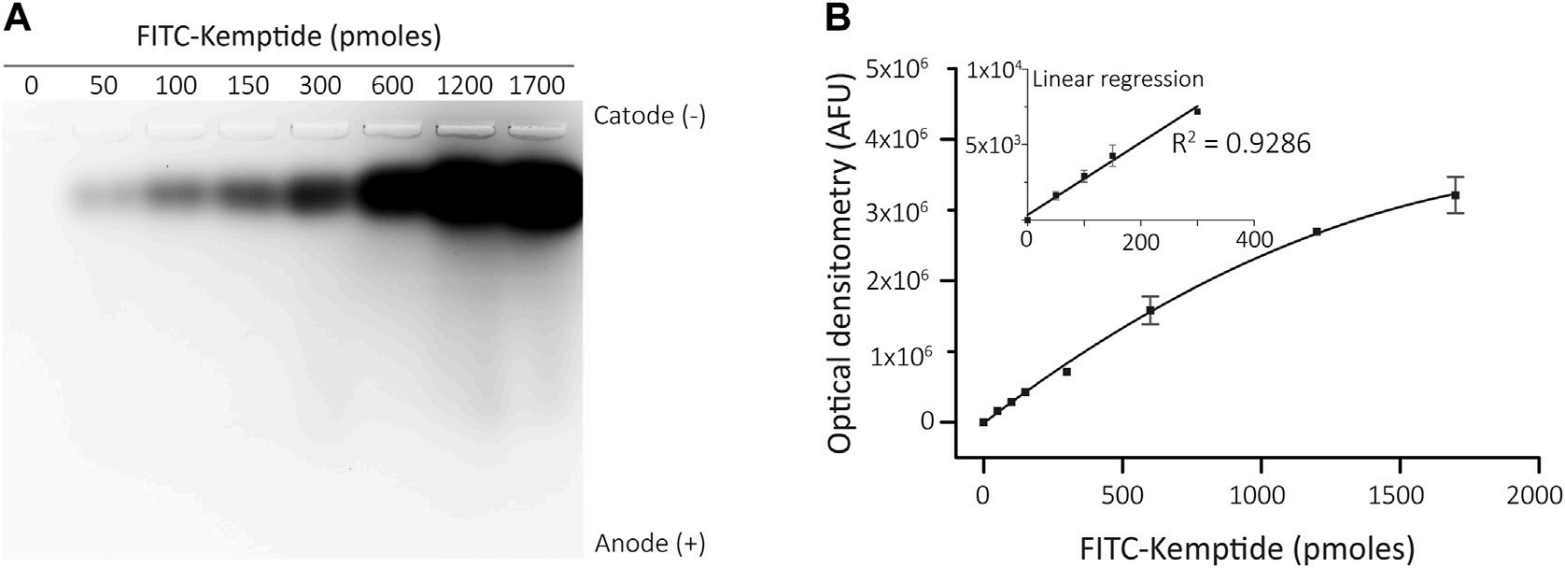
Linear range of Kemptide-FITC optimization. **(A)** Different amounts of FITC-conjugated Kemptide subjected to agarose gel electrophoresis, showing migration towards the anode. **(B)** FITC fluorescence quantifications (AFU, arbitrary fluorescence units). Insert shows a linear regression determined between 0-300 *p*moles Kemptide-FITC (*R* ^2^ = 0.9286).

To address the linearity of increasing fluorescence signals of FITC conjugated Kemptide, different amounts of the tagged peptide were loaded on a 1.5% agarose gel at pH 10. The non-phosphorylated Kemptide-FITC has net negative charge at pH 10, evidenced by migration towards the anode. The fluorescence signal was quantified by optical density and exhibited direct proportionality to the quantity of labelled peptide loaded. The linear response spanned from 50 to 300 *p*moles, with a linear regression coefficient of *R* ^2^ = 0.9286, saturation at 600 *p*moles and experimental detection limit of 50 *p*moles (Figure 1).

This analysis allowed us to set 300 *p*moles as an appropriate amount of Kemptide-FITC to be loaded on the agarose gels after the kinase reaction for our particular detection settings. Note that linearity values might change depending on the imaging settings and should be determined for each specific equipment.

### 3.2 KiMSA assay using recombinant PKA catalytic subunit

**FIGURE 2:**
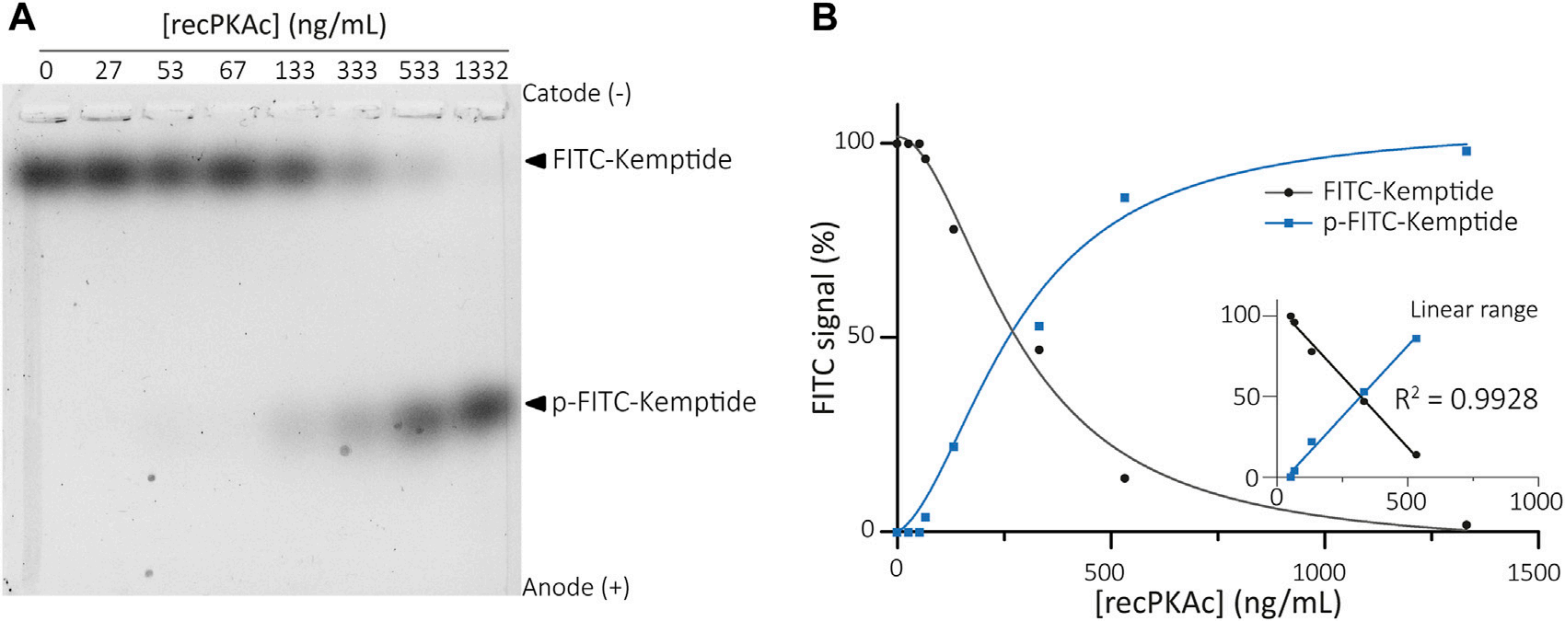
Recombinant PKAc activity assay. **(A)** Kinase reactions were performed using different concentrations of recPKAc. Mixtures were then analysed by agarose gel electrophoresis showing increasing amounts of Kemptide-FITC in its phosphorylated form upon increase of recPKAc. **(B)** FITC fluorescence quantifications (FITC signal (%) = F/Ft * 100, where F equals Kemptide-FITC or p-Kemptide-FITC band fluorescence intensity, and Ft equals total lane fluorescence intensity). Linear regression between [recPKAc] and FITC signal was determined between 53–533 ng/mL (graph inset, *R* ^2^ = 0.9928).

Different concentrations of recombinant catalytic PKA subunit (recPKAc) were used along with 60 µM of total Kemptide in kinase reactions. Enzymatic reactions were carried out at 37°C for 25 min and then resolved by agarose gels electrophoresis. As anticipated, two distinct signals were clearly observed migrating towards the anode. A low-mobility band corresponding to the non-phosphorylated Kemptide-FITC and a high-mobility band as the result of Kemptide phosphorylation (Figure 2). A linear response between 53 ng/mL and 533 ng/mL of recPKAc was observed at 37°C for a reaction time of 25 min (*R* ^2^ = 0.9974).

### 3.3 KiMSA assay for assessment of sperm PKA activity changes during capacitation

**FIGURE 3:**
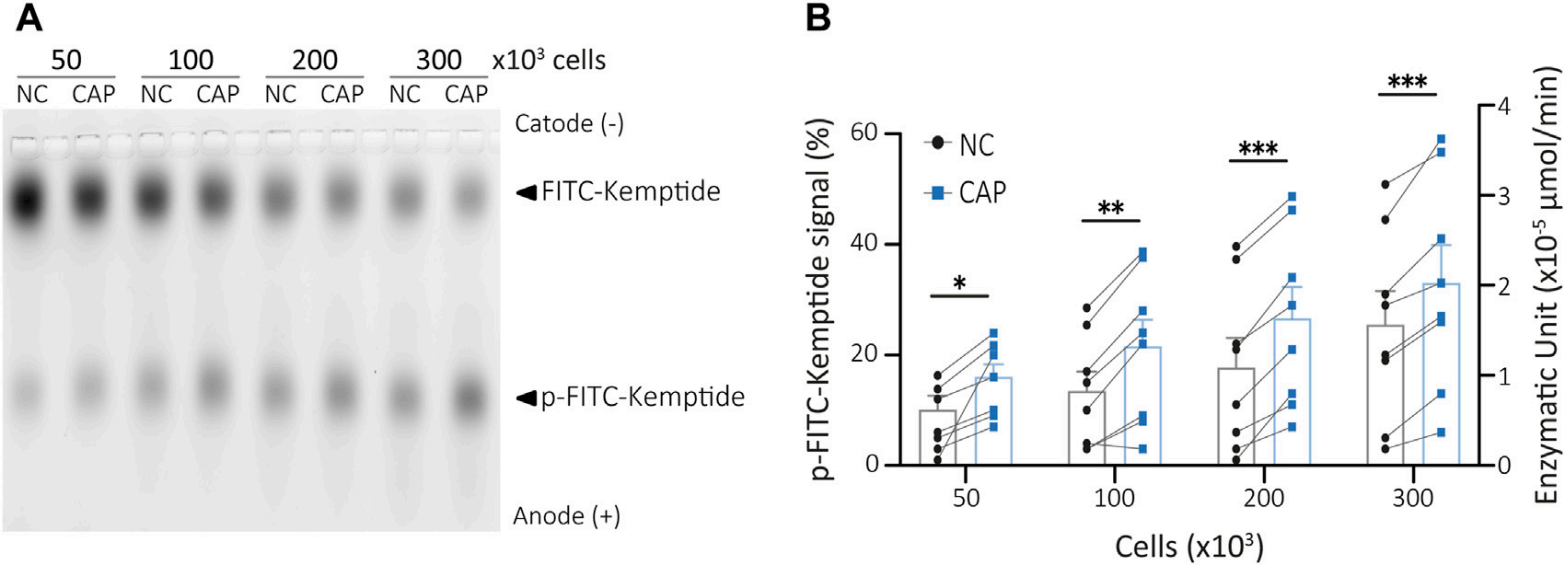
Sperm cells extract PKA activity assay. **(A)** Different amounts of capacitated or non-capacitated mouse sperm cells extract were used in kinase reactions and analysed in agarose gel electrophoresis. **(B)** p-Kemptide-FITC fluorescence quantifications (left axis, pKemptide-FITC signal (%) = F/Ft * 100, where F equals p-Kemptide-FITC band fluorescence intensity, and Ft equals total lane fluorescence intensity; right axis, Enzymatic units). NC, non-capacitating medium; CAP, capacitating medium. Paired two-tailed t-Student tests. *p < 0.05, **p < 0.01, ***p < 0.001. Data expressed as mean ± SEM, n > 7.

**FIGURE 4:**
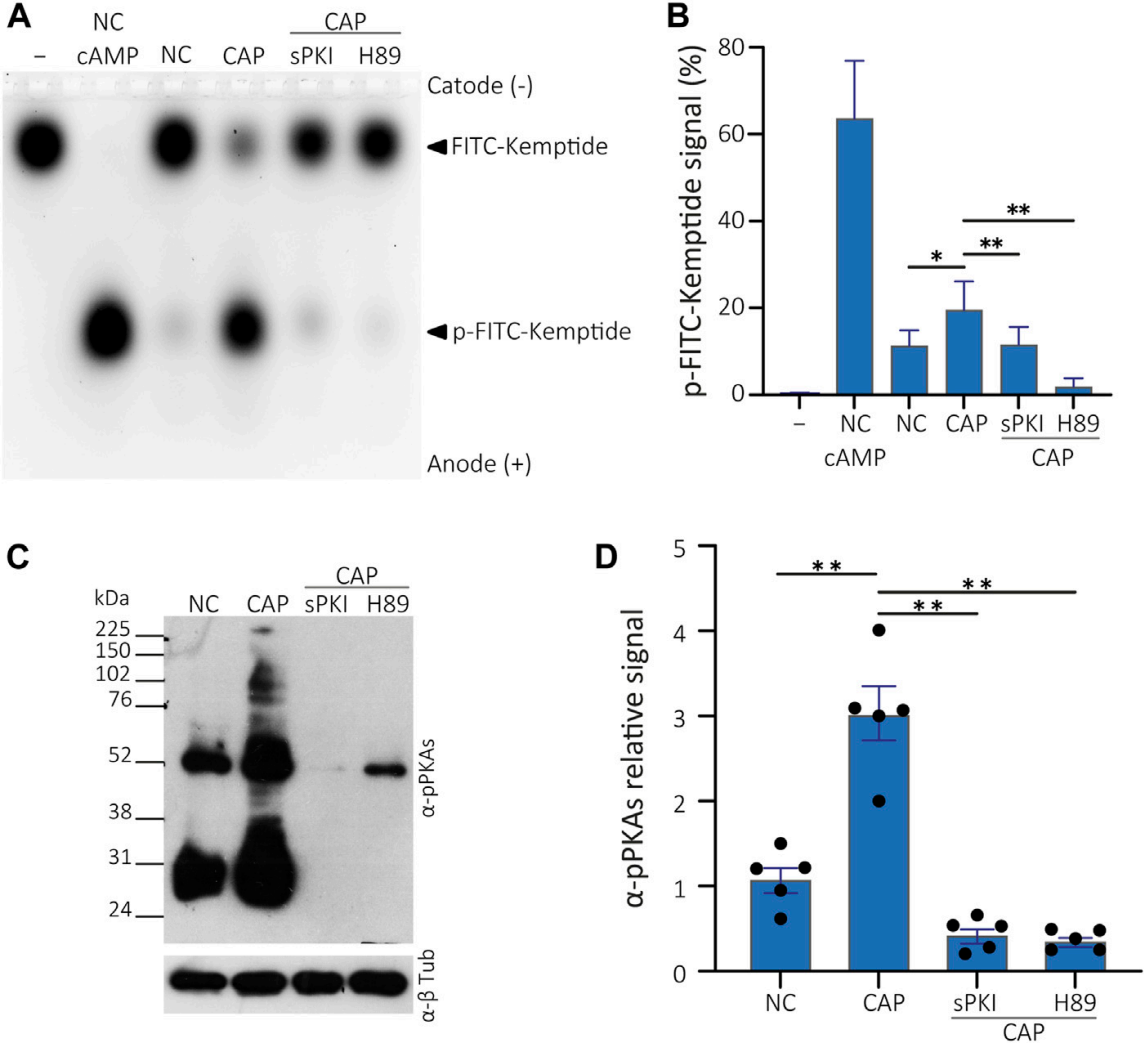
PKA activity assay in presence of PKA inhibitors. **(A)** Cells were incubated in non-capacitating conditions or in capacitating conditions containing or not either 15 µM sPKI or 30 µM H89. Sperm were further centrifuged and lysed for usage in kinase reactions subjected to agarose gel electrophoresis. Kemptide-FITC alone is shown as negative control (−) and non-capacitated mouse sperm extract used as positive control upon addition of 1 µM cAMP to kinase reaction mixture (+). **(B)** p-Kemptide-FITC fluorescence quantifications (pKemptide-FITC signal (%) = F/Ft * 100, where F equals p-Kemptide-FITC band fluorescence intensity, and Ft equals total lane fluorescence intensity). Matched one-way ANOVA, Tukey multiple comparison test. *p < 0.05, **p < 0.01, data expressed as mean ± SEM, n > 5. **(C)** Sperm were incubated for 15 min in non-capacitating or capacitating medium containing or not either 15 µM sPKI or 30 µM H89. Each condition was processed for Western blot analysis with a monoclonal anti-pPKAs antibody. Membrane was stripped and analysed for the presence of tubulin using anti-β-tub. **(D)** Densitometric analysis of pPKAs signals recovered from each whole lane, normalized against α-tubulin, of WB analysis shown in C (n = 5).

Most PKA activity assays are designed to detect the activity of either extract purified enzyme or recombinant PKA (Araujo et al., 2016; Stival et al., 2018; 2020). However, they do not reflect the state of PKA within the cells which depends on intracellular levels of cAMP. Four cAMP molecules bind to a pair of regulatory PKA subunits to release active PKAc (Taylor et al., 2013; Baro Graf et al., 2020). When analysing cellular PKA activity, it is then intended to maintain as much as possible the actual physiological state of the enzyme. To develop an assay that would reflect the activity of endogenous PKA under different treatments, the first step was to obtain cell lysates to be used for kinase reactions, as described in the previous section. As recently shown, maximal PKA activity during capacitation is achieved at 15 min of incubation in capacitating conditions (Baro Graf et al., 2020; Balbach et al., 2021). To assess this physiological activation of PKA by KiMSA, mouse sperm cells were incubated in either non-capacitating or capacitating media for 15 min. Extracts from 50, 100, 200 or 300 × 10^3^ sperm cells in final kinase reaction volumes of 25 µL were used to optimize the amount of starting cells. All tested cell extracts showed significant differences in PKA activity between sperm incubated in non-capacitating versus capacitating media, as the amount of phosphorylated Kemptide (lower bands) increased upon capacitation. As expected, the higher the number of cells used in the kinase reaction, the more phosphorylated Kemptide-FITC in both types of media. From all tested quantities, 300 × 10^3^ sperm cells was selected as starting material to be used in the kinase reaction protocol for quantitation of native sperm PKA activity, based on improved signal differences (Figures 3A, B, see also Figure 4). However, our results show that this assay can quantitatively and reproducibly address both basal and stimulated PKA activity in different sperm cells concentrations.

To discard the effect of kinases other than PKA acting on the Kemptide-FITC, cells were incubated with either 15 µM sPKI or 30 μM H89, two different PKA inhibitors with different modes of action. Inhibitors were present during incubation in either non-capacitating or capacitating media. Then, cells were centrifuged, and inhibitors washed away as standard protocol was followed, as explained above, to obtain total cell extracts. Kinase reactions were performed without further addition of inhibitors, showing impaired *in vitro* PKA activity (Figures 4A, B). Thus, KiMSA reflects the effect of *in vivo* incubation rather than the effect of inhibitors added to the reaction mixture. Note that differences that raised from capacitation of sperm cells in terms of PKA activity (NC and CAP lanes, Figures 4A, B), as well as when extracts deriving from NC cells were challenged with 1 µM cAMP (Figures 4A, B, lane NC cAMP). In parallel to KiMSA, equally treated cell samples were analysed for PKA activity through traditional Western blot detection of phosphorylated PKA substrates (pPKAs, Figures 4C, D), as an indirect marker of PKA activity in sperm (Krapf et al., 2010)**.** The results showed that the Kemptide-FITC substrate is not phosphorylated in the presence of PKA inhibitors. sPKI is considered as highly specific and allows discarding promiscuous phosphorylation effects by other kinases (Liu et al., 2020). Furthermore, KiMSA was able to reflect the *in vivo* status of PKA, since inhibitors were added only to the incubation media and removed before lysis.

### 3.4 KiMSA assay to quantify cAMP

**FIGURE 5:**
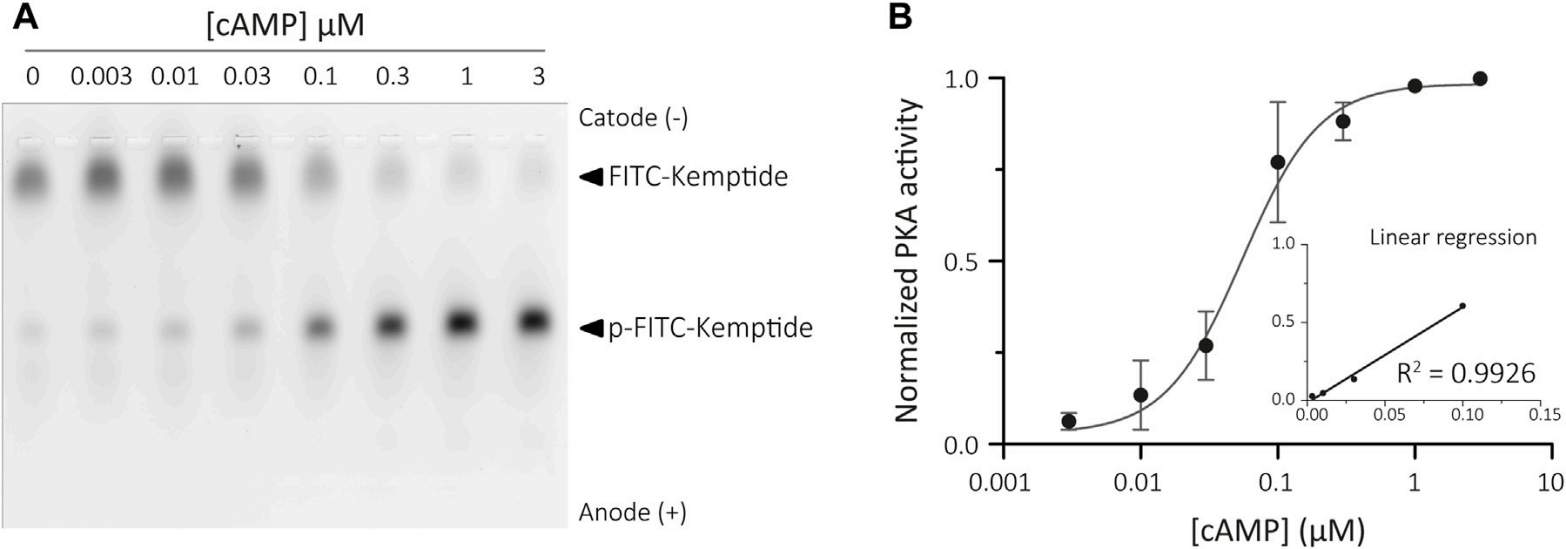
Calibration curve for cAMP assay. **(A)** Agarose gel electrophoresis of Kemptide-FITC incubated in non-capacitated mouse sperm cells extract containing different amounts of exogenous cAMP, showing increasing pKemptide-FITC amounts. **(B)** Densitometric analysis of PKA activity is shown for known concentrations of cAMP (normalised PKA activity = F/Ft * 100, where F equals p-Kemptide-FITC band fluorescence intensity, and Ft equals total lane fluorescence intensity). Linear regression curve between [cAMP] and PKA activity was determined for the range 3–100 nM cAMP (graph inset, *R* ^2^ = 0.9926).

Traditional activation of PKA relies on cAMP binding to the regulatory subunits. These regulatory subunits are tethered to Triton X-100 insoluble structures both in somatic (Bramson et al., 1982; Mucignat-Caretta and Caretta, 2001) and sperm cells (Vijayaraghavan et al., 1997; Visconti et al., 1997; Baker et al., 2007). Binding of cAMP results in a conformational change that releases the active catalytic subunits of PKA, which can then be collected in the soluble fraction. Therefore, inactive PKA holoenzyme can be recovered from the insoluble sperm fraction and remains sensitive to cAMP levels. This allowed us to adapt the KiMSA assay to indirectly measure the amounts of cAMP by assessing the extent of PKA activation recovered from the insoluble fraction of sperm cells when challenged with unknown concentrations of cAMP. When assessing cAMP levels in cellular extracts, holoenzyme is prepared fresh the day of the experiment, and a concentration curve using known amounts of cAMP needs to be constructed for every assay. The resulting plot is used to determine intracellular cAMP levels in sperm cells by interpolation (Wertheimer et al., 2013). To this end, sperm cells were immediately lysed in Triton X-100 buffer after swim-out, and the insoluble fraction was recovered to use in KiMSA experiments (see Materials for further details). The kinase reaction was performed over different known concentrations of cAMP in a final volume of 25 µL and the samples analysed in an agarose gel (Figure 5A). The PKA activity values obtained were used to generate a standard curve on a logarithmic scale (Figure 5B).

This method was able to reproducibly detect cAMP concentrations ranging from 3 nM to 100 nM, equivalent to 0.12–4 *p*moles (in 25 μL). This detection scale is optimized for detection of cAMP in mouse sperm samples (and other mammalian species), compared to the reported values of intracellular cAMP in mouse sperm cells ranging from 0.5-1 *p*moles in 1 × 10^6^ sperm cells incubated in non-capacitating medium to 1–3 *p*moles upon capacitating (Balbach et al., 2021).

## 4 Discussion

Given the ubiquity of PKA across various cell types and its pivotal involvement in diverse signalling pathways, the availability of tools for assessing its activity is of paramount importance. The study of PKA’s *in vitro* activity implies working with at least partially purified enzyme. This classic *in vitro* approach allows testing the direct effect of drugs (such as inhibitors or activators), as well as to analyse how certain post translational modifications or mutations might impact on the enzyme’s activity. Worth stating, having a reliable system that can reflect the state of PKA *in vivo* is crucial to study physiological events such as sperm capacitation.

In the context of sperm cells, two primary methodologies are commonly used to assess PKA activity. The first involves the analysis of the phosphorylation extent of cellular substrates of PKA through Western blot experiments (Visconti et al., 1997; Stival et al., 2018). To this end, sperm are incubated under different experimental conditions to later analyse cell extracts by Western blot using commercially available antibodies against the consensus phosphorylation sequence of PKA (RRXpS/pT) (Krapf et al., 2010). Although this approach is simple, caution should be taken when directly correlating PKA activity to substrates phosphorylation increase, since the phosphorylation status of any given protein within the cell depends on the relative activities of both kinases and phosphatases acting on it, as well as other indirect mechanisms of regulation such as subcellular localization of PKA (Baro Graf et al., 2020). In this sense, the disruption of PKA binding to AKAP anchoring proteins in sperm cells using the permeable peptide sHT31, resulted in complete suppression of phosphorylation of PKA substrates assessed by Western blot, suggesting inhibition of PKA activity (Stival et al., 2018). However, when PKA is exposed to sHT31 *in vitro,* the enzyme retained its catalytic activity, clearly demonstrating that the two approaches yield different outcomes (Vijayaraghavan et al., 1997; Stival et al., 2018). Therefore, care should then be taken when concluding on PKA activity using Western blot analysis disregarding the role of the compartmentalization of signalling domains and the dynamic modulation of other kinases and phosphatases. A more suitable approach, usually used in somatic cells for studying compartmentalized PKA activity, relies on the design of fluorescent reporters of PKA that track phosphorylation *in cellulo*. These procedures need transgenic cell lines and are mostly based on FRET emission changes of PKA synthetic substrates (Zhang et al., 2001). However, since the sperm is a terminally differentiated cell with no synthesis of *the novo* proteins (Stival et al., 2016), transgenesis techniques constitute a major challenge.

A second method to study PKA using sperm cells, involves directly measuring the activity of PKA *in vitro* by quantification of ^32^P transferred from [γ-32P]-ATP to the synthetic substrate Kemptide (Kemp et al., 1977; Stival et al., 2020)**.** This approach is performed in controlled media supplemented with phosphatase inhibitors, isolated from other factors that could affect the phosphorylated state of the substrate. The assay is suitable for analysing the direct effect of agonists or antagonists on PKA’s *in vitro* activity using recombinant PKA (Wertheimer et al., 2013; Stival et al., 2018). Alternatively, the inactive PKA holoenzyme could be isolated from the insoluble fraction of sperm cells or somatic cells (MacAla et al., 1998; Taskén et al., 2001; Araujo et al., 2016), for *in vitro* analyses**.** The major drawbacks of working with [γ-32P]-ATP include its short half-life, high cost, hazardous radioactivity, and the need for specialized equipment and handling procedures due to its emission of beta particles.

**FIGURE 6:**
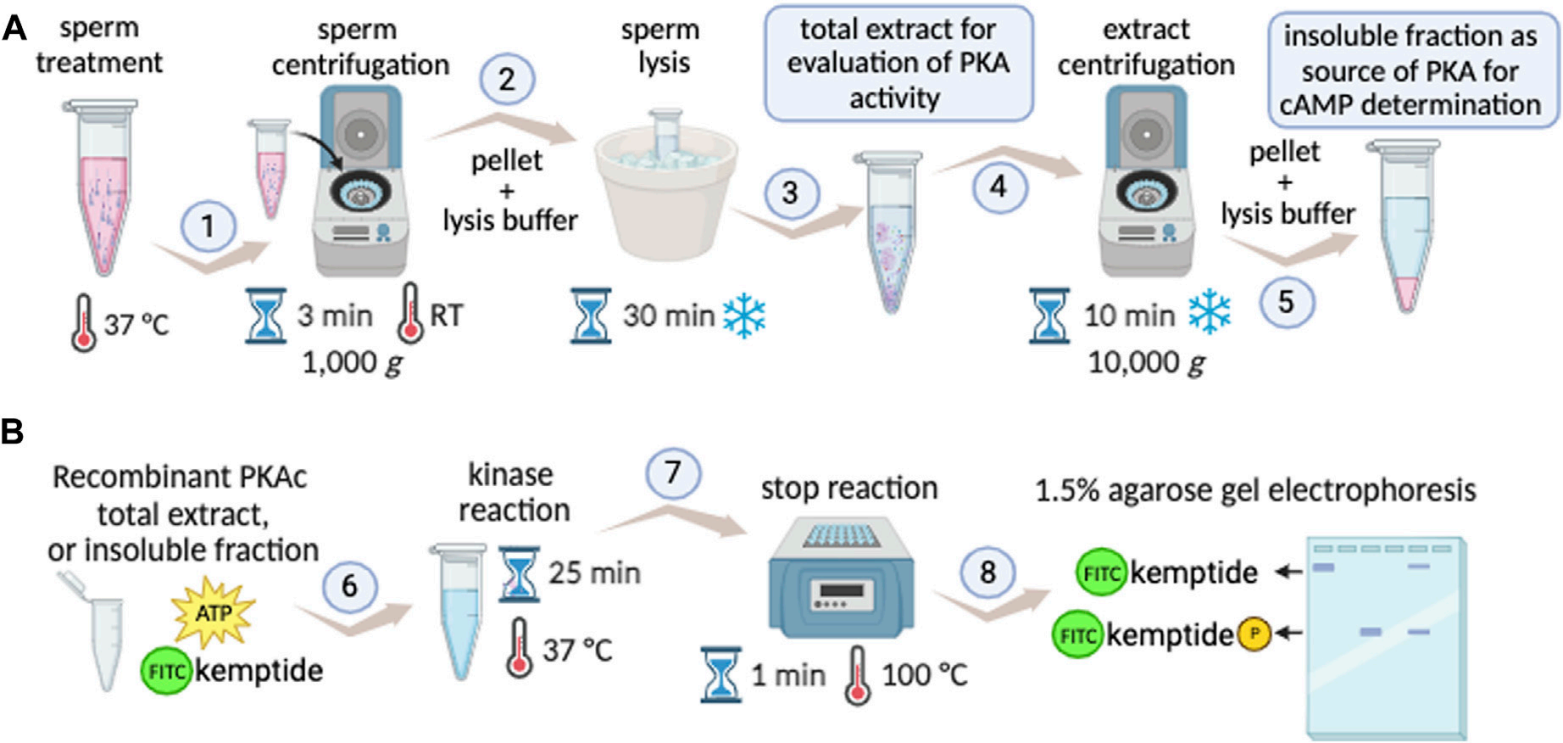
KiMSA workflow summary. **(A)** Diagram showing preparation steps for cell extracts obtention to either PKA activity evaluation during capacitation, or insoluble fraction as a source of PKA holoenzyme. Sperm cells are incubated according to experimental conditions to be assayed and cells are then pelleted at RT for 3 min at 1,000 g (1). Lysis buffer is added to the pellet and incubated on ice for 30 min (2). If total sperm extract is needed, it can be readily used (3) or further centrifuged at 4°C for 10 min at 10,000 g, for holoenzyme semi-purification (4). The insoluble fraction is used as holoenzyme (5). **(B)** Kinase assay, using either recombinant PKAc (recPKAc), total cell extract or insoluble fraction of sperm extracts is used, with kinase buffer as indicated for 25 min at 37°C (6). Reactions were stopped by incubation for 25 min at 100°C (7), and further analysed by agarose gel electrophoresis and densitometry of phospho-kemptide and kemptide signals (8).

The Kinase Mobility Shift Assay (KiMSA) standardized herein, is a versatile non-radioactive adaptation of the PKA activity assay, which instead of using ^32^P relies on fluorescent tagging of the Kemptide, retaining high sensitivity. The protocol is straightforward and can be easily deployed using standard equipment, as detailed in Figure 6. KiMSA can be used to address *in vitro* activity of PKA, to assess the state of activation of PKA in sperm extracts and to determine cellular cAMP concentrations.

KiMSA assay relies on FITC fluorescence to detect phosphorylated Kemptide and it is based on the ability to separate through electrophoresis the non-phosphorylated from the phosphorylated versions of it (Lutz et al., 1994; Araujo et al., 2016). Thus, the extent of the kinase reaction can be quickly determined by running an agarose gel electrophoresis. Then, the quantitation of the phosphorylated peptide can be done using basic laboratory equipment such as a fluorescence imaging system.

Besides avoiding the disadvantages previously mentioned for the radioactive methods, KiMSA assay is highly specific even in complex cell extracts, as the phosphorylation of Kemptide is highly PKA-specific. In addition, since the method only detects the tagged Kemptide as opposed to ^32^P that can be carried over to the mix, noise is significantly reduced.

In the sperm physiology field, PKA displays a key role as an important modulator for capacitation events (Baro Graf et al., 2020). Simple and robust methods for PKA analysis have been elusive for years for reproductive biologists. KiMSA has the potential to bridge this gap, both in sperm as well as in any other cell types. However, not only PKA is of great interest, but also intracellular 3′-5′-cyclic adenosine monophosphate (cAMP), one of the main second messengers involved in cell signalling (Buffone et al., 2014). Different techniques are available for cAMP detection, including: 1) FRET-based sensors (originally named FlCRhR) to track the extent of association between the regulatory and catalytic subunits of PKA in the presence of cAMP (Adams et al., 1991), 2) radioimmunoassays (RIA) that use immobilized anti-cAMP antibodies and ^125^I-labeled cAMP as a tracer molecule (Williams, 2004) and 3) enzyme-linked immunosorbent assay (ELISA)-based kits (Wertheimer et al., 2013). These methods have important drawbacks. The first one relies on recombinant fluorescein-labelled catalytic subunit and a rhodamine-labelled regulatory subunit of PKA, resulting in the need of transgenesis of the cell of interest to introduce the recombinant proteins. Modifications of this technique, involve EPAC-based cAMP sensors (DiPilato et al., 2004), and the sensor tested in sperm using FRET-based built from the cyclic nucleotide binding domain (CNBD) of the bacterial *Mloti*K1 channel (Mukherjee et al., 2016). On the other side, RIA and ELISA commercial kits offer the possibility to address cAMP concentrations in cell extracts. However, these procedures imply the use of antibodies which could derive in false results due to insufficient blocking or antibody instability with sometimes both non-reproducible results and high costs (Sakamoto et al., 2018). Conversely, KiMSA assay is a straightforward technique which yields reproducible results in the detection of cAMP amounts, in a range compatible to variations cAMP found during mouse and human sperm capacitation.

While we introduced and validated KiMSA for the quantitation of cAMP and the analysis of PKA activity, it can be readily customized for evaluating the activity of other kinases, provided there exists an available specific peptide substrate for each target enzyme. In addition, it could be used to screen for drugs that interfere with cAMP production in sperm for male contraceptive porpoises. The development of a reliable and safe method to measure PKA activity in sperm cells not only facilitates the investigation of physiological processes in which this kinase is involved but also offers a potential screening approach to explore the underlying causes of idiopathic male infertility associated with impaired sperm PKA activation. Moreover, this versatile tool can be applied to explore PKA pathways across diverse cellular systems, encompassing both normal physiological processes and disease states.

## Data Availability

The original contributions presented in the study are included in the article/Supplementary Material, further inquiries can be directed to the corresponding authors.
